# Correlation Among Phenotypic Parameters Related to the Growth and Photosynthesis of Strawberry (*Fragaria* × *ananassa* Duch.) Grown Under Various Light Intensity Conditions

**DOI:** 10.3389/fpls.2021.647585

**Published:** 2021-06-10

**Authors:** Hyo Gil Choi

**Affiliations:** Department of Horticulture, Kongju National University, Yesan, South Korea

**Keywords:** abnormal weather, chlorophyll fluorescence, image sensing, light, smart farm control

## Abstract

The objective of this study was to investigate characteristics of phenotypic parameters such as physiology, yield, and fruit quality responses of strawberry (*Fragaria* × *ananassa* Duch.) to various light intensity conditions (VLICs), and to determine the correlations among these phenotypic parameters. Strawberry plants were cultivated in a smart greenhouse separated into four areas, three of which were completely shaded by curtains from 20:00 until 10:00 (3 hS), 12:00 (5 hS), and 14:00 (7 hS), respectively. The fourth area was a non-shaded control treatment (0 hS). The ambient light intensities during the experimental period for the 0, 3, 5, and 7 hS treatments were 1,285, 1,139, 770, and 364 mol⋅m^–2^, respectively. Strawberry plants grown under low light intensity conditions experienced decreases in photosynthetic rate, stomatal conductance, and sugar accumulation compared to the 0 hS. Petiole generation and fruit yield were also sharply decreased in proportion to the degree of decrease in light intensity. In contrast, photosynthetic pigment content was shown to increase under low light conditions. Organic acid contents (excluding acetic acid) and leaflet size did not change significantly under low light conditions compared to the 0 hS. Changes to light intensity are considered to induce changes to the phenotypic characteristics of strawberry plants to favor growth using the energy and carbon skeletons obtained through respiration and photosynthesis. In the 7 hS treatment, where light intensity was drastically reduced, NPQ, qP, and R_*Fd*_ values as chlorophyll *a* fluorescence parameters were significantly lowered, which could indicate their measurement as an important technique to check the stress response of plants grown in low light conditions.

## Introduction

Various environmental factors are important for plant growth, such as light, temperature, carbon dioxide, and nutrients. Among these, light is particularly important, as it initiates photosynthesis through the photo-dissociation of water. Decreasing hours of sunlight in winter can negatively affect plant growth. In Korea, where there are many mountainous regions, the average number of hours of sunlight during the winter season (January and February) varies greatly from 2.3 to 8.0 h, depending on the region (Moon et al., 2016). Most plants experience many fluctuations in sunlight from full sun to shade throughout the day. Under these conditions, stomatal and photosynthetic responses vary dramatically related with growth status (Knapp and Smith, 1990). Horticultural crop growth and harvest are greatly reduced in areas with low winter sunlight intensity. Though not common, building infrastructure for social overhead capital, such as railroads and overpass construction, can block sunlight from reaching surrounding horticultural crop cultivation areas. Correspondingly lowered yields can then lead to lawsuits between the government and farmers (Lee et al., 2012). Zhu et al. (2017) indicated that low light is a pervasive abiotic stress in plant breeding and cultivation. In addition, the occurrence of abnormal sunlight conditions arising from global warming can have negative effects on crop cultivation (Suzuki et al., 2014; Raza et al., 2019). Generally, higher levels of light than are necessary for crop growth cause photostress, leading to photoinhibition, which reduces growth and decreases the efficiency of the quantum yield in reaction centers (RCs) of photosystem II (PSII) (Szymańska et al., 2017; Lima-Melo et al., 2019). In contrast, low light conditions during the cultivation of field crops and protected horticultural crops have been reported to not only reduce photosynthesis and plant growth, but also cause yield loss and quality degradation (Yu et al., 2016; Gao et al., 2017; Zhu et al., 2017; Lu et al., 2019).

Strawberry (*Fragaria* × *ananassa* Duch.) is a horticultural crop in the family Rosaceae that is cultivated around the world and can be grown under low-temperature conditions. Strawberries are produced commercially in 76 countries, followed by China, United States, Mexico, Turkey and Spain as the top five producing nations (Simpson, 2018). Strawberry is a popular fruit-vegetable and functional food its excellent activities and potential health benefits (Giampieri et al., 2014). The growth of strawberry plants in greenhouses is worldwide a widespread cultivation method to provide a suitable environment conditions to cultivate as excellent functional food (Tang et al., 2020). In Korea, strawberry production has reached to 208,699 tons in 2017 and the total area of production is about 6,435 (Wei et al., 2020) and strawberry plants are usually grown in greenhouses from September through April. However, in recent decades, there has been much variation in ambient light intensity during the cultivation period in different regions, resulting in some regions experiencing serious issues with strawberry growth due to insufficient light (Lee et al., 2020).

Many methods are available to confirm the physiological plant growth responses to various environmental conditions. Because plants are photoautotrophs and use photosynthesis for growth, parameters such as photosynthetic rate, stomatal conductance, and transpiration rate are often used (Zuo et al., 2018; Zheng et al., 2019). Chlorophyll *a* (Chl *a*) fluorescence has also been used to determine crop growth under different environmental conditions, as this is a non-destructive method for diagnosing plant stress through the photo-physiological response. Values that are frequently used as parameters of Chl *a* fluorescence include the minimal fluorescence when all PSII RCs are open (*F*_*o*_), the maximal fluorescence when all PSII RCs are closed (*F*_*m*_), the maximum quantum yield of PSII photochemistry (*F*_*v*_/*F*_*m*_), non-photochemical quenching of maximal fluorescence (NPQ), the fluorescence decline ratio under a given light condition (R_*Fd*_), and photochemical quenching of variable fluorescence (qP) (Choi et al., 2016; Calvo et al., 2017; Wang et al., 2017). The accumulation of substances in the fruits of strawberry plants also varies greatly depending on the environment (Cervantes et al., 2019; Talukder et al., 2019). Soluble sugars can be easily used as a respiration source for the production of energy, while organic acids are used as precursors to various secondary metabolites (Pant et al., 2015). Therefore, analyzing the levels of these substances in the fruit is an important method for assessing the quality of horticultural crops grown under various environmental conditions.

When growing commercial strawberries, the lack of light intensity for various reasons can significantly reduce productivity and deteriorate quality of fruit. In terms of supplemental light under lack of light intensity, a number of recent studies have focused effects of LED light to increase productivity and quality of strawberry fruit (Talukder et al., 2018; Kepenek, 2019; Zheng et al., 2019). However, studies on the photo-physiological properties, yield and fruit quality of strawberry according to different light intensity levels are rare. Therefore, the aim of this study was to confirm the correlation among phenotypic parameters, such as photosynthetic rate, stomatal conductance, transpiration rate, and Chl *a* fluorescence of the leaves, and the yield and quality of strawberry fruits in response to various light intensity conditions (VLICs) adjusted by artificial shading.

## Materials and Methods

### Plant Cultivation and Materials

**FIGURE 1:**
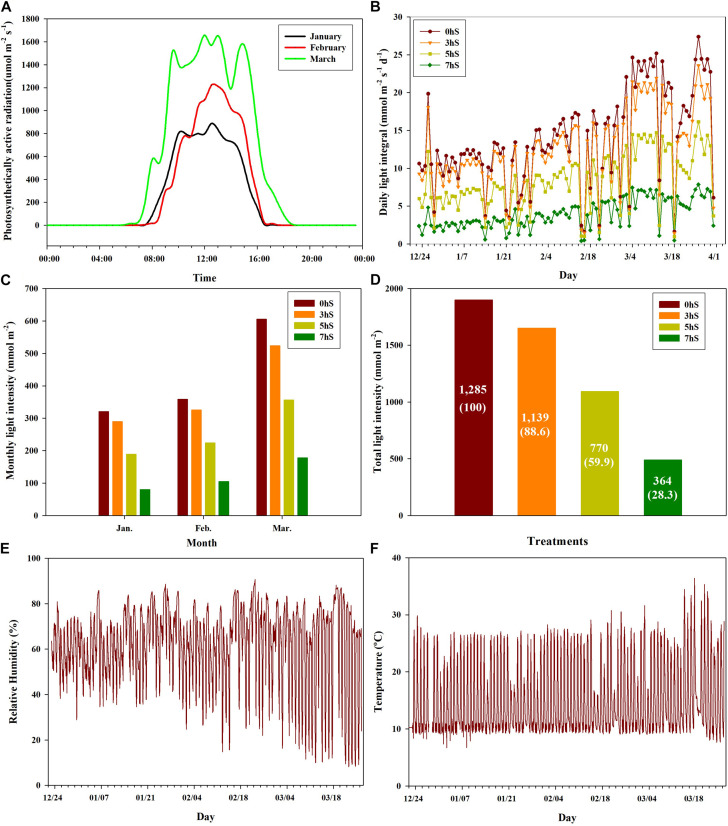
Ambient light intensities under different shading treatments and aerial environmental conditions in a smart greenhouse used for strawberry (*Fragaria* × *ananassa*) cultivation. **(A)** distribution of light intensity on sunny days by month; **(B)** daily light integral by month; **(C)** monthly light intensity; **(D)** total light intensity during the experimental period; **(E)** relative humidity inside the smart greenhouse from January to March 2019; **(F)** temperature inside the smart greenhouse from January to March 2019. 0 hS, non-shading treatment (control); 3 hS, three hours of shading until 10:00; 5 hS, five hours of shading until 12:00; 7 hS, seven hours of shading until 14:00.

Strawberry plant seedlings (*Fragaria* × *ananassa* Duch. “Selhyang”) were planted in a high-floor bench bed system filled with a commercial medium (Tosille Medium; Shinan Grow Co., Jinju, South Korea) in a smart greenhouse (automatic control using Wi-Fi communication) at Kongju National University, South Korea. The smart greenhouse was divided into four areas using an automated opening and closing curtain system, and the strawberry plants (seedlings age around 65 days) were planted on October 24, 2018. During cultivation, the plants were supplied with water and a nutrient solution [Research Station for Floriculture and Glasshouse Vegetables (PBG) nutrient solution; macro-elements N:P:K:Ca:Mg:S = 12.5:3.0:5.5:6.5:2.5:3.0 me⋅L^–1^; micro-elements Fe:B:Mn:Zn:Cu:Mo (1.12:0.27:0.55:0.46:0.05:0.05 mg⋅L^–1^; electrical conductivity (EC) = 1.0 dS⋅m^–1^; hydrogen ion concentration (pH) (5.5–6.0)] via a drip irrigation system at 2-min intervals, up to five times per day. The greenhouse temperature was controlled by heating when the ambient temperature was below 10°C, and by opening ventilators when the temperature was above 25°C. Photosynthetically active radiation levels were recorded at 1-h intervals using LI-190 quantum sensors (Licor, NE, United States) installed 100 cm below the curtain parallel to the height of the strawberry plants at the central position in each of the four areas of the greenhouse. Light levels in the four areas were controlled using automatic 100% shading curtains, which were closed at 20:00 each day and sequentially opened in three of the areas at 10:00, 12:00, and 14:00, respectively. The fourth area was left unshaded as a control. Strawberry plants were acclimated in the same environmental conditions for a period of 60 days after planted and then the shading treatments were applied from December 23, 2018 until March 31, 2019. During the experiment periods, the sunrise times were 07:20 in late-December, 07:30 in January, 07:10 in February and 06:40 in March. The approximate sunrise time from December to March after shading treatments was 07:10. Thus, four light intensity conditions were created in the greenhouse for this experiment: non-shading as a control (0 hS), three hours of shading until 10:00 (3 hS), five hours of shading until 12:00 (5 hS), and seven hours of shading until 14:00 (7 hS). The total light intensity according to the shading treatment of 0, 3, 5, and 7 hS was 1,285 mol⋅m^–2^, 1,139 mol⋅m^–2^, 770 mol⋅m^–2^, 364 mol⋅m^–2^, respectively (Figure 1).

### Analysis of Photosynthesis and Chlorophyll Fluorescence

Photosynthesis and Chl *a* fluorescence parameters were measured in nine different 20-day-old strawberry leaves per treatment from February 6–10, 2019. In addition, the photosynthetic rate, stomatal conductance, and transpiration rate of the leaves were measured in the smart greenhouse using a portable photosynthesis system (LI-6800; Licor, NE, United States) on clear mornings (08:00–10:00). The chamber conditions of the LI-6800 were set as follows: chamber flow 500 μmol⋅s^–1^, chamber overpressure 0.1 kPa, fan speed 10,000 rpm, RH 50%, photon flux density 1,000 μmol⋅m^–2^⋅s^–1^, chamber temperature 25°C, and CO_2_ 400 μmol⋅mol^–1^.

To analyze the pulse-amplitude-modulated Chl *a* fluorescence, strawberry leaves were harvested at 7:00 on a clear day and sealed in a dark bottle, then swiftly moved to the laboratory to minimize water stress, and subjected to measurement with a fluorometer (FluorCam FC 800; Photon Systems Instruments, Drasov, Czechia) after 20-min of dark adaptation.

### Analysis of the Photosynthetic Pigment, Soluble Sugars, Organic Acid Contents

Strawberry leaves and fruits were harvested on January 24, February 26, and March 24, 2019. Photosynthetic pigment content of the leaves and soluble sugar and organic acid contents of the fruit were measured for each treatment. To analyze the photosynthetic pigment content, six leaf disks (1 g fresh weight) were punched out of each leaf (30 ± 5 days old) with a cork borer and macerated in 15 mL of acetone (containing 100 mg of CaCO_3_) with a homogenizer (PT-3100; Kinenatica AG, Switzerland). The homogenate was then poured into a solvent-resistant microfuge tube, spun for 5-min, and the resulting supernatant was collected. The extract was filtered and the absorbance was measured at 661.6, 644.8, and 470 nm with a UV-VIS spectrophotometer (Evolution 300; Thermo Fisher Scientific, MA, United States), as described by Lichtenthaler (1987):

Chlorophylla=(11.24×A.661)6-(2.04×A.644)8

Chlorophyllb=(20.13×A.644)8-(4.19×A.661)6

Carotenoids=(1000×A)470-(1.90×Chla-63.14×Chlb)/214

To analyze the soluble sugar and organic acid contents, strawberry fruits were crushed into fruit juice using a homogenizer (PT-3100; Kinenatica AG, Switzerland) and each crushed extract was centrifuged at 16,000 *g* for 30 min at 4°C (64R Centrifuge; Beckman Coulter Inc., CA, United States). The supernatant was then filtered through Whatman No. 2 filter paper and immediately frozen and stored at -70°C. Prior to analysis, the frozen samples were defrosted and filtered through 0.45 μm syringe filter.

The soluble sugars were then analyzed with a high-performance liquid chromatography system (YL9100; Younglin Co., Anyang, South Korea) equipped with a Sugar-Pak (4.6 mm × 240 mm, Supelco, PA, United States) column and RI detector (YL9170, Younglin Co., Anyang, South Korea). The separation was conducted at 30°C with the mobile phase of acetonitrile: water (75:25, v/v) at a flow rate of 1 mL⋅min^–1^. The identification of the sugars in the fruits was completed by comparing the retention times of the individual sugars in the reference vs. tested solution. Several carbohydrates, such as fructose, glucose, and sucrose, were quantitatively assayed. The contents of these compounds were calculated by comparing the pear areas obtained in the examined samples with those from the reference solution, as described by Choi et al. (2015).

The organic acid contents in the extracts of the fruits were analyzed with an ion chromatography system (ICS 5000, Dionex, CA, United States) equipped with Ion-Pac column (9 mm × 250 mm ICE-AS6, Dionex, NY, United States) and a suppressor (AMMS ICE300, Dionex, NY, United States). The mobile phase was 0.4 mM heptaflurobutyric acid and the flow-rate was 1 mL⋅min^–1^. The anion self-regenerating suppressor was provided with 5.0 mN tetrabutyl ammonium hydroxide and 5.0 psi N_2_ in recycle mode to reduce eluent background conductivity. Oxalic acid, citric acid, and malic acid were identified by comparing their spectra with those of standards. Total organic acid content was calculated by combining the respective amount of those three acids, according to Choi et al. (2015).

### Yield and Aerial Growth Measurement

Fully ripe fruits were harvested at weekly intervals from January through March to determine the yield of each treatment group. Aerial growth, including the number of petioles, petiole length, leaflet length, leaflet width, and crown diameter, was measured using a microcaliper (Mitutoyo 500, Mitutoyo Co., Tokyo, Janpan) on the last Wednesday of each month from December 2018 through March 2019.

### Experimental Design and Statistical Analysis

This experiment used a randomized block design that included four blocks, each comprising 30 plants grown using a high bench bed system with hydroponics. There were three replicates per block. Photosynthesis and chlorophyll fluorescence parameters were measured in nine replicates (plants) per treatment, while all other parameters were analyzed in three replicates (blocks) per treatment. Results related with phytochemicals are expressed as the mean ± standard deviation of three measurements. To confirm differences among the treatment groups, the data were analyzed using one-way analysis of variance with Duncan’s multiple range test using a significance level of *p* ≤ 0.05 in SAS (SAS Institute Inc., NC, United States). Phenotypic parameters coefficients of correlation were analyzed using Pearson’s correlation in SAS software.

## Results

### Environments Condition in the Smart Greenhouse

The ambient light intensities of the 0, 3, 5, and 7 hS treatments administered in a smart greenhouse are shown in Figure 1. The distribution of light intensity on sunny days from January through March is also shown (Figure 1A). In the smart greenhouse, daytime lasted approximately 8 h in January and February, and approximately 12 h in March. During the 99-day treatment period, there were 81 days of clear weather and 18 days of cloudy weather (Figure 1B). The amount of light entering the smart greenhouse was approximately twice as high in March (606 mol⋅m^–2^) as in January (320 mol⋅m^–2^) (Figure 1C). The 3, 5, and 7 hS treatments received total light intensity levels of 88.6, 59.9, and 28.3%, respectively, of the 0 hS (1,285 mol⋅m^–2^) level of 100% (Figure 1D). The nighttime relative humidity in the greenhouse was 75–85% during the whole experiment periods, but the daytime relative humidity was around 35% until mid-February, and dropped sharply after mid-February (Figure 1E). During the experimental period, the nighttime temperature in the greenhouse was maintained at around 8°C. The daytime temperature was lowest in January and highest in March, when some days were over 30°C (Figure 1F).

### Changes in Phenotypic Parameters Related Photosynthesis

**FIGURE 2:**
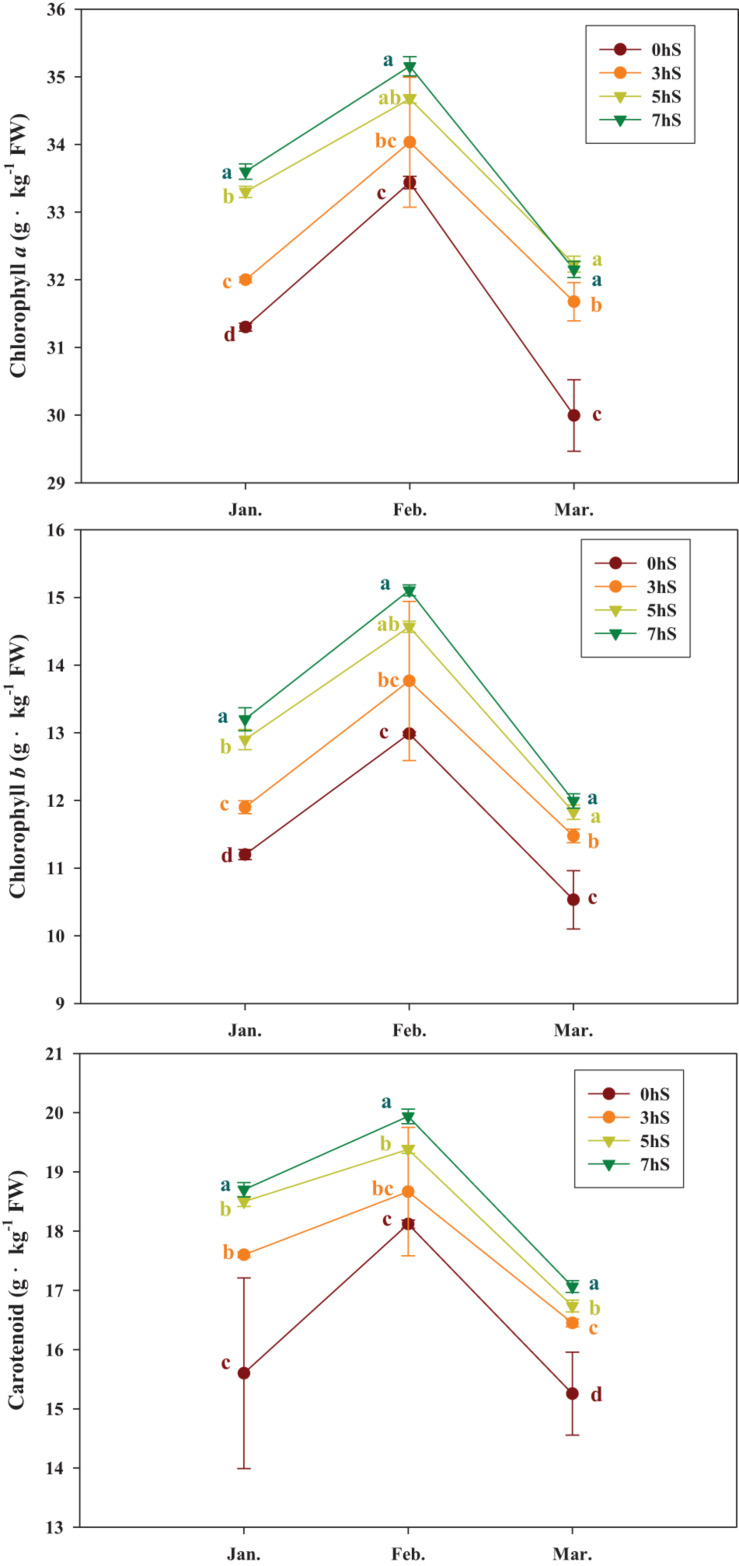
Photosynthetic pigment contents of strawberry (*Fragaria* × *ananassa*) leaves (30 ± 5 days old) grown under various light intensity conditions in a smart greenhouse. Vertical bars represent standard deviations. Different letters above the bars indicate significant differences among treatments (Duncan’s multiple range test, *p* < 0.05, *n* = 3). 0 hS, non-shading treatment (control); 3 hS, three hours of shading until 10:00; 5 hS, five hours of shading until 12:00; 7 hS, seven hours of shading until 14:00. FW, fresh weight.

Strawberry leaf contents of the photosynthetic pigments Chl *a*, chlorophyll *b* (Chl *b*), and carotenoids changed under VLICs (Figure 2). In all treatments, the photosynthetic pigment content was higher in February than in January or March. However, the photosynthetic pigment content of the leaves in March was significantly reduced in the 0 hS compared to the other treatments. Overall, photosynthetic pigment content increased as light intensity decreased; however, there was a significant decrease in the photosynthetic pigment content when the light intensity fell to the level of 5 hS. The Chl *a*, Chl *b*, and carotenoid contents of the strawberry leaves across the various treatments ranged from 29–35, 10–16, and 14–20 g⋅kg^–1^, respectively.

**FIGURE 3:**
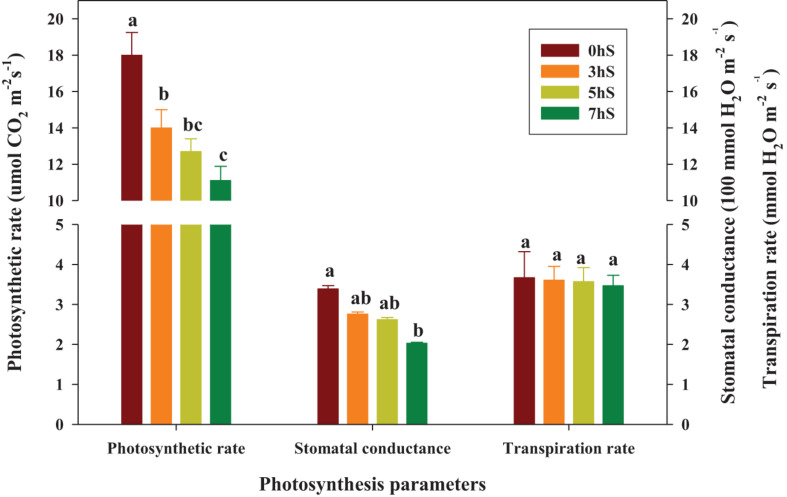
Photosynthesis parameters of strawberry (*Fragaria* × *ananassa*) leaves grown under various light intensity conditions in a smart greenhouse. Vertical bars represent standard deviations. Different letters above the bars indicate significant differences among treatments (Duncan’s multiple range test, *p* < 0.05, *n* = 9). 0 hS, non-shading treatment (control); 3 hS, three hours of shading until 10:00; 5 hS, five hours of shading until 12:00; 7 hS, seven hours of shading until 14:00.

Values of the photosynthetic rate, stomatal conductance, and transpiration rate in strawberry leaves grown under VLICs are shown in Figure 3. The photosynthetic rate of the strawberry leaves was 29, 42, and 62% higher under 0 hS conditions (18 μmol CO_2_⋅m^–2^⋅s^–1^) than under 3, 5, and 7 hS, respectively. Similarly, the stomatal conductance of the leaves was 23, 29, and 67% higher under 0 hS conditions (3.39 100 mmol H_2_O⋅m^–2^⋅s^–1^) than under 3, 5, and 7 hS, respectively. However, transpiration rate did not differ significantly among treatments.

**FIGURE 4:**
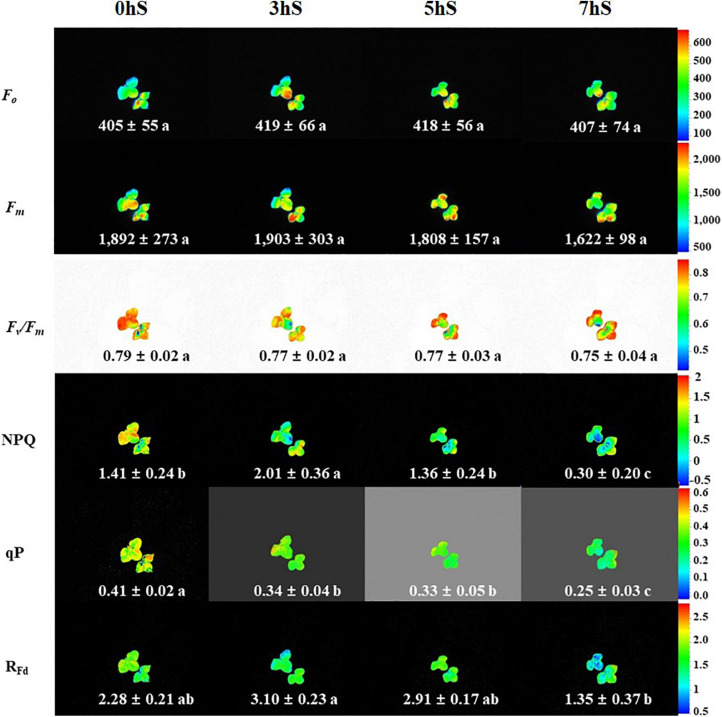
Chlorophyll *a* fluorescence of strawberry (*Fragaria* × *ananassa*) leaves grown under various light intensity conditions in a smart greenhouse. 0 hS: non-shading treatment (control); 3 hS, three hours of shading until 10:00; 5 hS, five hours of shading until 12:00; 7 hS, seven hours of shading until 14:00; *F*_*o*_, minimum fluorescence when all photosystem II (PSII) reaction centers (RCs) are open; *F*_*m*_, maximal fluorescence when all PSII RCs are closed; *F*_*v*_/*F*_*m*_, maximum quantum yield of PSII photochemistry; NPQ, non-photochemical quenching of maximal fluorescence; qP, photochemical quenching of variable fluorescence; R_*Fd*_, fluorescence decline ratio in light. Average values included standard deviation followed by different lower-case letters within a column are significantly different (Duncan’s multiple range test, *p* < 0.05, *n* = 9).

To confirm the state of strawberry leaves grown under VLICs, Chl *a* fluorescence was measured as an indicator of stress (Figure 4). There was no significant difference in the *F*_*o*_ and *F*_*m*_ values of the strawberry leaves under VLICs. There was likewise no significant difference in *F*_*v*_/*F*_*m*_ values, which ranged from 0.79 to 0.75, under VLICs. However, NPQ, qP, and R_*Fd*_ showed significant differences among treatments, with NPQ and R_*Fd*_ being higher under 3 hS (2.01 and 3.10, respectively) than under the 0, 5, and 7 hS treatments. The qP was significantly higher in leaves grown under 0 hS conditions as 0.4 ± 0.02 than under the three shading treatments. In addition, the values of all three parameters fell sharply under 7 hS compared with the other treatments.

### Changes in Growth of the Aboveground Part of Plant

**FIGURE 5:**
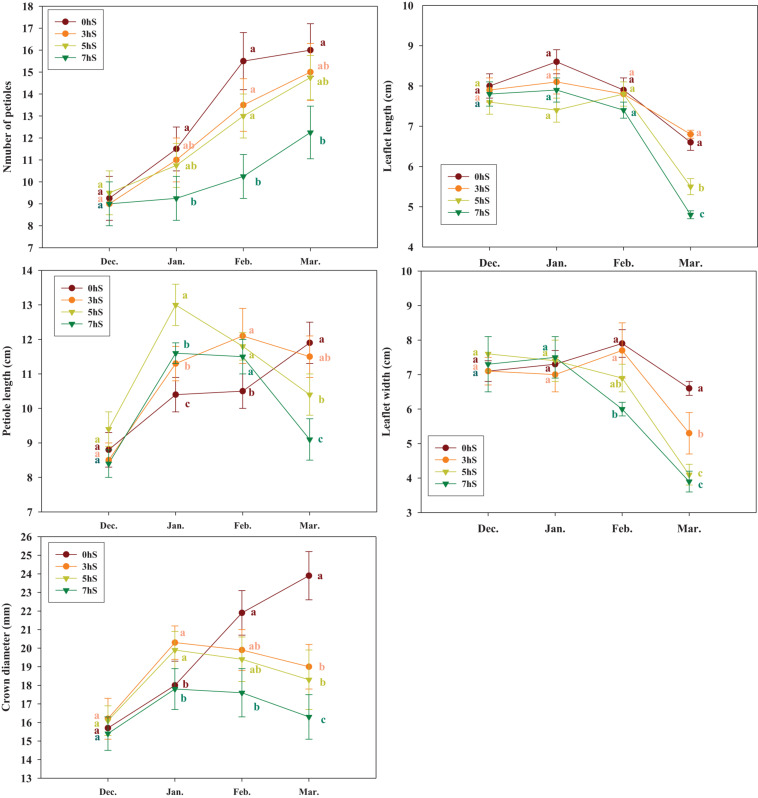
Growth of aboveground part of the strawberry (*Fragaria* × *ananassa*) plants grown under various light intensity conditions in a smart greenhouse. Vertical bars represent standard deviations. Different letters above the bars indicate significant differences among treatments (Duncan’s multiple range test, *p* < 0.05, *n* = 3). 0 hS, non-shading treatment (control); 3 hS, three hours of shading until 10:00; 5 hS, five hours of shading until 12:00; 7 hS, seven hours of shading until 14:00.

Figure 5 shows changes in the growth of the aboveground part of strawberry plants from before the experiment began in December, and then in January, February, and March while grown under VLICs. In December, the strawberry plants had consistent number of petioles, petiole length, leaflet length, leaflet width, and crown diameter, with no significant differences detected among the treatment groups. However, after treatment with VILCs, the number of petioles and crown diameter were significantly lower in plants grown under 7 hS. In addition, in March, when the difference in the light intensity according to shading treatment was wider (Figure 1C), the growth of the aboveground part of plants under 0 and 7 hS showed a very significant statistical difference (Figure 5). Crown diameter of plants in the 0 hS steadily increased with increasing light intensity from December to March. However, under 3, 5, and 7 hS, crown diameter only increased until January, and then decreased in February and March.

### Commercial and Non-commercial Fruit Yield

The yields of commercial (single fruit weight (10 g) and non-commercial (single fruit weight < 10 g and malformed fruit) strawberry fruit produced under VLICs are shown in Tables 1, 2. From January to March, the average commercial-grade fruit weight was 22 g and the total commercial fruit yield was 482 g per plant under 0 hS conditions. Commercial yields under the 3, 5, and 7 hS treatments were 69, 43, and 24% lower than the 0 hS, respectively. The weight of commercial fruit was also found to increase proportionally with increasing light intensity. In contrast, the non-commercial fruit yield increased as light intensity decreased, with 70% more non-commercial fruit being harvested under 7 hS compared with the 0 hS group. Non-commercial fruit harvested in the 0 hS was less than 10% of the commercial fruit, but the proportion of non-commercial fruit increased as the light intensity decreased. Under 7 hS, the non-commercial fruit yield was over 50% greater than the commercial fruit yield.

**TABLE 1:** Commercial fruit yield of strawberry (*Fragaria* × *ananassa*) grown under various light intensity conditions in a smart greenhouse.

Treatment	Commercial fruit yield (g*cdot*plant^–1^)											
	January			February			March			Total		
	Yield	AW	Ratio	Yield	AW	Ratio	Yield	AW	Ratio	Yield	AW	Ratio
0 hS	49 ± 1.5a^*z*^	14.4 ± 0.2a	100	85 ± 1.2a	23.5 ± 0.2a	100	348 ± 13.0a	23.3 ± 0.5a	100	482 ± 16.6a	22.0 ± 0.3a	100
3 hS	51 ± 1.3a	15.5 ± 0.2a	105	71 ± 2.7b	18.2 ± 0.2b	84	208 ± 14.6b	20.8 ± 1.1b	60	330 ± 13.1b	19.2 ± 0.6b	69
5 hS	34 ± 3.2b	16.0 ± 0.6a	69	43 ± 0.5c	14.3 ± 0.1d	51	131 ± 6.1 c	18.2 ± 0.2c	38	208 ± 7.7 c	16.9 ± 0.1c	43
7 hS	39 ± 1.6b	16.3 ± 0.6a	80	17 ± 0.1d	16.7 ± 0.0.1c	20	60 ± 3.7 d	15.4 ± 0.7d	17	116 ± 2.2 d	15.8 ± 0.3d	24

*^z^Average values included standard deviation followed by different lower-case letters within a column are significantly different (Duncan’s multiple range test, p ≤ 0.05, n = 3). Commercial fruit means fruit weighing more than 10 g. AW is the average fruit weight. Ratio refers to the percent yield of each treatment compared with the control. 0 hS: non-shading treatment (control); 3 hS, three hours of shading until 10:00; 5 hS, five hours of shading until 12:00; 7 hS, seven hours of shading until 14:00.*

**TABLE 2:** Non-commercial fruit yield of strawberry (*Fragaria* × *ananassa*) grown under various light intensity conditions in a smart greenhouse.

Treatment	Non-commercial fruit yield (g⋅plant^–1^)											
	January			February			March			Total		
	Yield	AW	Ratio	Yield	AW	Ratio	Yield	AW	Ratio	Yield	AW	Ratio
0 hS	–	–	–	8.0 ± 0.1c^*z*^	8.0 ± 0.7a	100	32.2 ± 2.7ab	8.3 ± 0.3a	100	33.0 ± 2.6 b	8.3 ± 0.2a	100
3 hS	–	–	–	20.4 ± 1.4ab	7.6 ± 1.0a	255	28.4 ± 1.3ab	6.3 ± 0.1a	88	48.8 ± 2.7ab	7.0 ± 0.3a	148
5 hS	–	–	–	15.3 ± 0.5b	9.6 ± 0.3a	191	37.2 ± 3.0 a	7.0 ± 0.2a	116	52.5 ± 3.4ab	7.5 ± 0.1a	159
7 hS	–	–	–	37.5 ± 1.9a	8.5 ± 0.2a	469	21.8 ± 1.0 c	6.4 ± 0.2a	68	59.3 ± 2.8 a	7.5 ± 0.1a	179

*^z^ Average values included standard deviation followed by different lower-case letters within a column are significantly different (Duncan’s multiple range test, p ≤ 0.05, n = 3). Non-commercial fruit means fruit weighing less than 10 g. AW is the average fruit weight. Ratio refers to the percent yield of each treatment compared with the control. 0 hS: non-shading treatment (control); 3 hS: three hours of shading until 10:00; 5 hS: five hours of shading until 12:00; 7 hS: seven hours of shading until 14:00.*

### Soluble Sugars and Organic Acid Contents of the Fruit

The soluble sugar contents of strawberry fruits grown under VLICs are shown in Table 3. Soluble sugar content was higher under the 0 and 3 hS conditions than under the 5 and 7 hS conditions in January and March, but not in February. In all 3 months, the soluble sugar content was significantly lower under 5 and 7 hS than under the 0 hS. Soluble sugar content was the highest in January and the lowest in March for all treatments excluding 3 hS in February.

**TABLE 3:** Soluble sugar contents of strawberry (*Fragaria* × *ananassa*) fruit grown under various light intensity conditions in a smart greenhouse.

Treatment	Soluble sugar content (g⋅100 g^–1^ fresh weight)											
	January				February				March			
	Fructose	Glucose	Sucrose	Total	Fructose	Glucose	Sucrose	Total	Fructose	Glucose	Sucrose	Total
0 hS	3.5 ± 0.2 a^*z*^	3.3 ± 0.4 a	2.1 ± 0.1a	8.9 ± 0.8a	2.6 ± 0.1a	2.4 ± 0.3 a	1.6 ± 0.3a	6.6 ± 0.4a	1.9 ± 0.1a	2.2 ± 0.1a	2.4 ± 0.1a	6.5 ± 0.4a
3 hS	3.2 ± 0.1ab	3.1 ± 0.2ab	2.0 ± 0.2a	8.3 ± 0.5a	2.3 ± 0.1b	2.1 ± 0.1ab	1.2 ± 0.1b	5.6 ± 0.1a	1.9 ± 0.1a	2.1 ± 0.2a	2.1 ± 0.1a	6.1 ± 0.6a
5 hS	3.1 ± 0.1ab	2.5 ± 0.3 b	1.4 ± 0.2b	7.0 ± 0.6b	2.1 ± 0.1b	1.9 ± 0.1 b	1.1 ± 0.1b	5.1 ± 0.1b	1.5 ± 0.1b	1.6 ± 0.2b	1.2 ± 0.1b	4.3 ± 0.4b
7 hS	2.2 ± 0.1 c	1.7 ± 0.1 c	1.3 ± 0.1b	5.2 ± 0.1c	2.3 ± 0.1b	1.9 ± 0.1 b	1.0 ± 0.1b	5.2 ± 0.1b	1.3 ± 0.1c	1.4 ± 0.1b	1.2 ± 0.1b	3.9 ± 0.4b

*^z^Average values included standard deviation followed by different lower-case letters within a column are significantly different (Duncan’s multiple range test, p ≤ 0.05, n = 3). 0 hS, non-shading treatment (control); 3 hS, three hours of shading until 10:00; 5 hS, five hours of shading until 12:00; 7 hS, seven hours of shading until 14:00.*

Unlike the soluble sugar content, the organic acid content of the fruit did not show a tendency to increase or decrease according to light intensity (Table 4). However, the organic acid content of the fruit was higher when the light intensity was highest in March. When the organic acids were separated into their components, the acetic acid content was higher in fruit grown under the 0 hS conditions in all harvested months, whereas the malic acid content tended to be highest in fruit grown under 7 hS in January and February when there was a lower light intensity.

**TABLE 4:** Organic acid contents of strawberry (*Fragaria* × *ananassa*) fruit grown under various light intensity conditions in a smart greenhouse.

Treatment	Organic acids contents (mg⋅100 g^–1^ fresh weight)								
	January			February			March		
	Citric acid	Malic acid	Acetic acid	Citric acid	Malic acid	Acetic acid	Citric acid	Malic acid	Acetic acid
0 hS	513 ± 86a^*z*^	179 ± 26ab	15.7 ± 5.8 a	494 ± 105a	164 ± 36a	22.5 ± 11.0a	607 ± 23 a	197 ± 64a	14.3 ± 1.7a
3 hS	484 ± 55 b	173 ± 54bc	14.2 ± 9.9ab	430 ± 56 a	152 ± 22a	12.2 ± 2.7 b	564 ± 56ab	195 ± 16a	11.3 ± 1.2b
5 hS	499 ± 40ab	167 ± 40 c	15.5 ± 8.9 a	527 ± 27 a	171 ± 14a	14.4 ± 1.7 b	542 ± 10ab	190 ± 20a	7.0 ± 0.2c
7 hS	495 ± 27ab	188 ± 28 a	11.2 ± 27.3b	454 ± 66 a	178 ± 23a	10.4 ± 1.7 b	502 ± 22 b	190 ± 21a	7.2 ± 0.2c

*^z^Average values included standard deviation followed by different lower-case letters within a column are significantly different (Duncan’s multiple range test, p ≤ 0.05, n = 3). 0 hS, non-shading treatment (control); 3 hS, three hours of shading until 10:00; 5 hS, five hours of shading until 12:00; 7 hS, seven hours of shading until 14:00.*

### Correlation Among Phenotypic Parameters

**FIGURE 6:**
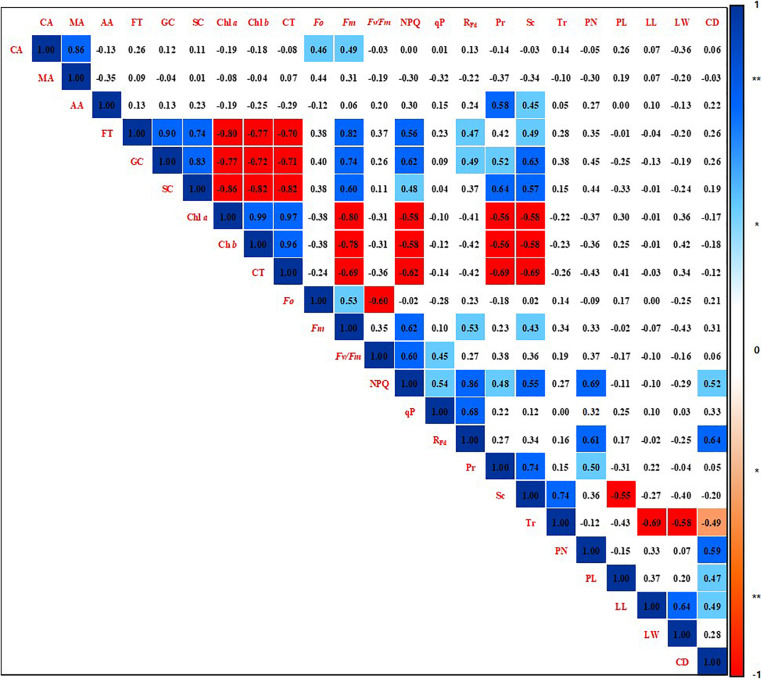
Correlation coefficients among phenotypic parameters of strawberry (*Fragaria* × *ananassa*) plants grown under various light intensity conditions in a smart greenhouse. Significant differences are shown at the 5 and 1% levels (*n* = 9), respectively, using Pearson correlation coefficients. **Dark blue box: positive correlation of 99%, **red box: negative correlation of 99%, *light blue box: positive correlation of 95%, *pink box: negative correlation of 95%, white box: non-significant correlation. CA, citric acid; MA, malic acid; AA, acetic acid; FT, fructose; GC, glucose; SC, sucrose; Chl *a*, chlorophyll *a*; Chl *b*, chlorophyll *b*; CT, carotenoids; *F*_*o*_, minimum fluorescence when all photosystem II (PSII) reaction centers (RCs) are open; *F*_*m*_, maximal fluorescence when all PSII RCs are closed; *F*_*v*_/*F*_*m*_, maximum quantum yield of PSII photochemistry; NPQ, non-photochemical quenching of maximal fluorescence; qP, photochemical quenching of variable fluorescence; R_*Fd*_, fluorescence decline ratio in light; Pr, photosynthetic rate; Tr, transpiration rate; Sc, stomatal conductance; PN, petiole number; PL, petiole length; LL, leaflet length; LW, leaflet width; CD, crown diameter.

A correlation analysis was performed among the phenotypic parameters of strawberry plants by calculating the Pearson product-moment correlation (Pearson, 1895; Figure 6). The correlation coefficient was a quantification using the analyzed data by all shading treatments for the overall experiment period. Citric acid was found to have a positive correlation with malic acid (*r* = 0.86, *p* < 0.01), *F*_*o*_ (*r* = 0.46, *p* < 0.05), and *F*_*m*_ (*r* = 0.49, *p* < 0.05). Acetic acid was positively correlated with photosynthetic rate (*r* = 0.58, *p* < 0.01) and stomatal conductance (*r* = 0.45, *p* < 0.05). The soluble sugars fructose, glucose, and sucrose not only showed with each other a positive correlation at a 99% significance level, but also had a high positive correlation with *F*_*m*_, NPQ, R_*Fd*_, photosynthetic rate, and stomatal conductance. On the other hand, soluble sugars were negatively correlated with the photosynthetic pigments Chl *a*, Chl *b*, and carotenoids at the 99% significance level. In addition, these photosynthetic pigments showed a high negative correlation with *F*_*m*_, NPQ, R_*Fd*_, photosynthetic rate, and stomatal conductance with 99% significance. The photosynthetic pigments also showed a high positive correlation at the 99% significance level reciprocally. NPQ was strongly positively correlated not only with parameters related to chlorophyll fluorescence, but also with photosynthetic rate (*r* = 0.48, *p* < 0.05), stomatal conductance (*r* = 0.55, *p* < 0.01), petiole number (*r* = 0.69, *p* < 0.01), and crown diameter (*r* = 0.52, *p* < 0.05). R_*Fd*_ showed a high positive correlation with petiole number (*r* = 0.61, *p* < 0.01) and crown diameter (*r* = 0.64, *p* < 0.01). Stomatal conductance showed a high positive correlation with photosynthetic rate (*r* = 0.74, *p* < 0.01) and transpiration rate (*r* = 0.74, *p* < 0.01), but there was no correlation between photosynthetic rate and transpiration rate. Photosynthetic rate was positively correlated with petiole number (*r* = 0.50, *p* < 0.05). Stomatal conductance was negatively correlated with petiole length (*r* = −0.55, *p* < 0.01). Transpiration rate was negatively correlated with leaflet length (*r* = −0.69, *p* < 0.01), leaflet width (*r* = −0.58, *p* < 0.01), and crown diameter (*r* = −0.49, *p* < 0.05). Phenotypic parameters related to the aerial parts of strawberry plants were not correlated with metabolites such as organic acids, soluble sugars, and pigments.

**FIGURE 7:**
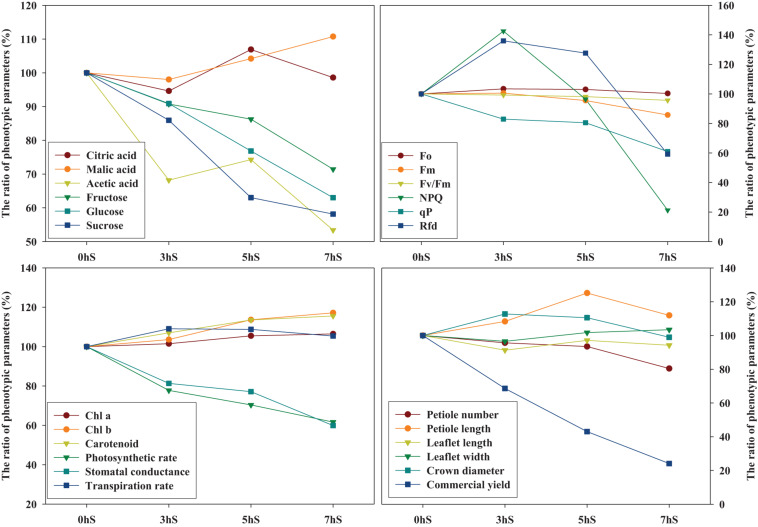
Relationship of graph patterns among phenotypic parameters of strawberry (*Fragaria* × *ananassa*) plants based on the 0 hS (100%). The graph pattern according to the ratio change of phenotypic parameters of strawberry plants for each light intensity level was used the statistically analyzed mean values in Tables 1, 3, 4, and Figures 2–5. Chl *a*, chlorophyll *a*; Chl *b*, chlorophyll *b*; *F*_*o*_, minimum fluorescence when all photosystem II (PSII) reaction centers (RCs) are open; *F*_*m*_, maximal fluorescence when all PSII RCs are closed; *F*_*v*_/*F*_*m*_, maximum quantum yield of PSII photochemistry; NPQ, non-photochemical quenching of maximal fluorescence; qP, photochemical quenching of variable fluorescence; R_*Fd*_, fluorescence decline ratio in light. 0 hS: non-shading treatment (control); 3 hS, three hours of shading until 10:00; 5 hS, five hours of shading until 12:00; 7 hS, seven hours of shading until 14:00.

The graph pattern according to the ratio change of phenotypic parameters of strawberry plants for each light intensity level was used the statistically analyzed mean values in Tables 1, 3, 4 and Figures 2–5 (Figure 7). Among the various phenotypic parameters, the sugar content of strawberry fruit, commercial fruit yield, and photosynthetic efficiencies of leaves such as photosynthetic rate and stomatal conductance linearly decreased as the light intensity reduced.

## Discussion

As photoautotrophs, plants grow by converting energy from the sun into their own energy source using photosynthetic pigments in the chloroplasts, such as Chl *a*, Chl *b*, and carotenoids. Consequently, changes in the chlorophyll contents of plants can indicate physiological responses to various environmental conditions. Chloroplasts can move to evade the short-term absorption of strong light, allowing them to avoid light-induced damage to PSII and any consequent photoinhibition (Kong et al., 2013; Szymańska et al., 2017). However, in the present study, changes in the Chl *a*, Chl *b*, and carotenoid contents were measured instead of chloroplast movement to determine the long-term effects of light intensity, rather than the temporary effects of different light levels. In this study, the Chl *a*, Chl *b*, and carotenoid contents of strawberry leaves continued to increase under low light intensity conditions in January and February, and decreased significantly as the light intensity reached its highest level in March (Figures 1, 2). It was determined that the photosynthetic pigment content of the leaves increased to efficiently absorb more light energy when grown under a low light intensity, similar to findings for *Physocarpus alternans*, which exhibits an increased chlorophyll content under long-term low light conditions (Zhang et al., 2016). Thus, it is thought that plant chlorophyll levels change to accommodate the continuous absorption of large amounts of light, as similarly reported by Feng et al. (2019).

During photosynthesis, carbon dioxide is fixed to produce sugars; therefore, photosynthetic rate can be used to investigate the physiological responses of plants to various environmental conditions (Korotaeva et al., 2018; Feng et al., 2019). Stomatal conductance and photosynthesis are linked by the ability of the stomata to enhance photosynthesis by their operation, while also avoiding dehydration induced damage (Hubbard et al., 2001). Under the conditions used in the present study, the photosynthetic rate and stomatal conductance of strawberry leaves increased proportionally with increasing light intensity (Figures 1, 3). This supports previous findings that increased stomatal conductance induces a rapid change in photosynthetic rate in response to light conditions (Yamori et al., 2020), and that there is a linear relationship between photosynthetic rate and stomatal conductance over a broad range of environmental conditions (Ball et al., 1987; Miner et al., 2017). Photosynthetic rate and stomatal conductance of strawberry leaves were also highly positively correlated (Figure 6). It is known that transpiration rate regulates the flow of carbon dioxide and its evaporation through the stomata, and is related to photosynthetic efficiency (Tuzet et al., 2003). In contrast, photosynthetic rate did not correlate with the transpiration rate of strawberry leaves under the VLICs examined in the present study (Figure 6), indicating that while there was a difference in the supply of carbon dioxide through the stomata, there was no significant difference in the transpiration of moisture through the stomata. It has previously been reported that crops show a large change in transpiration rate with changes in temperature (Ben-Asher et al., 2008). It was found that the experimental conditions in the present study, such as temperature and relative humidity, did not cause moisture stress in strawberry plants, and so did not affect the transpiration rate (Figure 1).

It is devised that photosynthetic rate is related to the dark reactions of photosynthesis, and Chl *a* fluorescence is related to the light reactions. Pulse-amplitude modulated Chl *a* fluorometry is related to photosynthetic efficiency and is widely used to confirm how efficiently plants can transport electrons in PSII under various environmental conditions. For most plants, the *F*_*v*_/*F*_*m*_ ratio is close to 0.83 in a stress-free environment, and lower than this under stressful conditions (Kalaji et al., 2017). In the present study, *F*_*v*_/*F*_*m*_ tended to decrease under low light intensity conditions, though these differences were not statistically significant (Figure 4). This finding is similar to previous reports on the stress levels of plants in different environments (Jamil et al., 2007; Choi et al., 2016). It has been reported that R_*Fd*_ can be used to determine the stress state of plants under high irradiance (Lichtenthaler et al., 2005), and that there are strong correlations between salt tolerance and Chl *a* fluorescence parameters such as NPQ and qP in tomato leaves (Zribi et al., 2009). In the present study, NPQ, qP, and R_*Fd*_ of the Chl *a* fluorescence reaction in the PSII RCs of strawberry leaves sharply decreased when grown under 7 hS compared with the other treatments, suggesting that strawberry plants are under low light stress when they receive a light intensity below 5 mmol⋅m^–2^⋅d^–1^ (Figures 1, 4). Yuan et al. (2017) reported that plants grown in low light conditions have decreased stomatal conductance and photosynthesis, as well as restricted electron transport in the chloroplasts, leading to a stressful state. NPQ has been shown to be positively correlated with photosynthetic rate and stomatal conductance in VLICs (Figure 6), which could make it an important parameter for confirming the stress state of plants in extremely low light conditions.

The aerial growth of strawberry plants differed significantly among treatments beginning in February, after 1 month or more of the shading treatment had occurred. These results indicate that crown diameter growth in strawberry plants is very closely related to light intensity, deteriorating when the light intensity is below a certain level, and that the growth of strawberry leaves is also proportionally related to light intensity (Figure 5). Kim et al. (2004) reported that light conditions have a large influence on the growth of chrysanthemum plantlets. Similarly, Fletcher et al. (2002) found that the aerial growth of strawberry plants was reduced with increasing shading. However, Demirsoy et al. (2007) did not observe this effect. Therefore, it seems likely that plants respond in different ways to create an optimal leaf shape under a given set of environmental conditions.

In this study, it confirmed that the commercial fruit yield of strawberries increased proportionally with an increase in light intensity (Table 1). In contrast, the non-commercial fruit yield increased proportionally with decreased light intensity (Table 2). The commercial fruit yield in our study matches previous reports that likewise found a positive correlation between light intensity and strawberry fruit yield (Choi et al., 2016). This is because strawberry plants grown in low light intensity do not achieve sufficient photosynthesis, causing decreased synthesis of soluble sugars such as glucose, fructose, and sucrose, which are necessary for fruit development.

The accumulation of soluble sugars in the fruit was affected by not only light intensity, but also seasonal factors. The soluble sugar content of the strawberry fruits was much lower in fruit harvested in March than in January. This was caused by increasing temperatures as the season changed from winter to spring (Table 3; Figure 1), supporting previous findings that increased growing temperature results in decreased soluble sugar content in strawberry fruits (Wang and Camp, 2000).

Unlike the soluble sugar content, the fruit organic acid content increased from winter to spring, likely due to increased temperature. According to Ruiz-Nieves et al. (2021), the organic acid content of tomatoes increased corresponding to rising greenhouse temperature. These traits are important in evaluating the quality of strawberry fruit; the combination of low light intensity and high temperature leads to fruit with decreased soluble sugar content and increased organic acid content, resulting in poor taste.

In plants, the distribution of energy produced by photosynthesis is described as sink and source relationship. As the light that plants can absorb decreases, photosynthesis eventually decreases; this is highly correlated with decreased sources of soluble sugars, which are products of photosynthesis. Conversely, as light intensity decreases, the photosynthetic pigment content increases; these pigments are negatively correlated with the photosynthetic rate, which is understood as an increase in the pigment content to allow more light absorption (Figure 6). Similar findings have been reported in the literature about lettuce grown under different light intensity condition (Fu et al., 2012). The contents of citrate and malate, two intermediates in the Krebs cycle of cell respiration, did not change significantly even when the light intensity was reduced, indicating that their abundance was not correlated with photosynthesis (Figure 6). Maintenance respiration is considered to remain constant, even when photosynthesis is reduced under low light intensity conditions. Strawberries that were grown under an environment with reduced light intensity compared to the 0 hS showed a sharp decrease in soluble sugar content, acetic acid content, petiole length, photosynthetic rate, and stomatal conductance. On the other hand, there were no significant changes in chlorophyll content and organic acid content (Figure 7).

**FIGURE 8:**
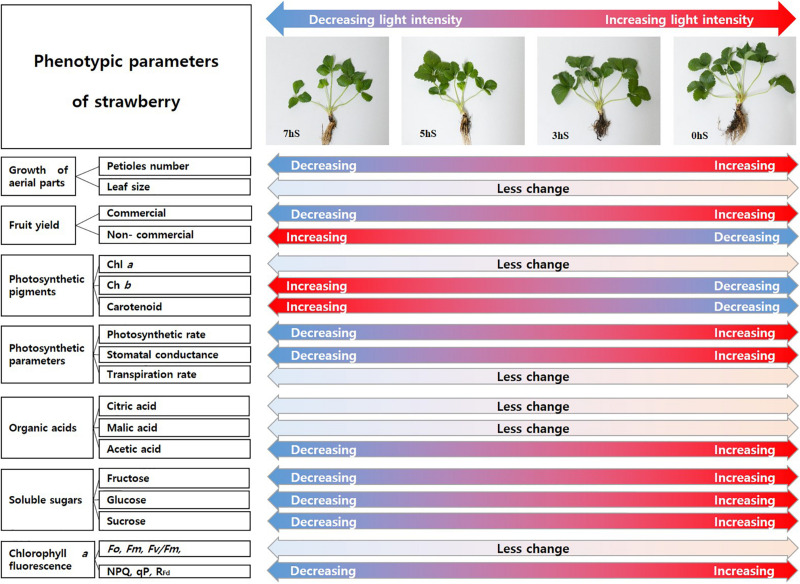
Schematic diagram of the degree of changes of phenotypic parameters of strawberry (*Fragaria* × *ananassa*) plants according to light intensity. 0 hS, non-shading treatment (control); 3 hS, three hours of shading until 10:00; 5 hS, five hours of shading until 12:00; 7 hS, seven hours of shading until 14:00.

According to Finkemeier and Sweetlove (2009), malate is a central metabolite of the plant cell with important roles in plant physiology and metabolism and is controller in plant homeostasis. This literature on the malate homeostasis in plants is explained to support this result in which malic acid remained steady without decreasing under low light intensity conditions. In terms of sink and source relationship for the distribution of soluble sugars produced by photosynthesis of strawberries grown under VLICs, these results are thought to induce changes to phenotypic characteristics in favor of growth using energy and carbon skeletons obtained through cell respiration and photosynthesis (Figures 7, 8). Future studies will be required to ascertain the correlation among the growth, production, and metabolic homeostasis by plants under VLICs.

Considering the phenotypic change due to energy distribution of strawberry plants under VLICs (Figure 8), plants choose whether to use the energy synthesized through photosynthesis to produce more biomass or to increase fruit yields or to maintain basic homeostasis. It is confirmed if the light intensity is insufficient, the strawberry plant consumes a relatively large amounts of energy to synthesize photosynthetic pigments, absorbing more light and maintain basic biomass levels. It is believed that sustaining current life is more important to plants than increasing fruit yields in the future. Eventually, when the ambient light intensity increases to the light saturation point, it can be expected to increase fruit yield and improve quality in strawberry plants. However, the recent ambient environment is changing unfavorably to the cultivation of horticultural crops. In recent studies, when artificial light sources are used for crop cultivation, it has been reported that the yield of crops can be increased (Kozai, 2016; Gómez and Izzo, 2018; Bambara and Athienitis, 2019). Therefore, it is judged that it is effective to use an artificial light source up to the level of obtaining an economic effect when growing strawberry.

In conclusion, as a result of analyzing changes in the phenotypic characteristics of strawberry plants grown under light intensity conditions in the ranging of 5–25 mol⋅m^–2^⋅d^–1^, I would like to state the following 3 points: (1) This study confirms that the change in light intensity did not have a significant effect on the leaflet size and organic acid content of strawberry plants, but had a positive correlation in photosynthetic rate, petiole formation, fruit yield, and sugar content. On the other hand, there was a negative correlation in photosynthetic pigments such as Chl *b* and carotenoid; (2) It is that NPQ, qP, and R_*Fd*_ values in Chl *a* fluorescence parameters can be suggested as an important indicator to confirm stress state of horticultural crops under low light intensity condition; (3) It is proposed to use a supplemental light source within the range of economic effect in order to stably produce crops under insufficient light intensity condition in a greenhouse.

## Data Availability Statement

The original contributions presented in the study are included in the article/supplementary material, further inquiries can be directed to the corresponding author/s.

## Author Contributions

HC planned the study, conducted the experiments, analyzed the data, and wrote the manuscript.

## Conflict of Interest

The author declares that the research was conducted in the absence of any commercial or financial relationships that could be construed as a potential conflict of interest.

## Abbreviations

Chl *a*: chlorophyll *a*
Chl *b*: chlorophyll *b*
*F*_*o*_: minimal fluorescence when all PSII RCs are open
*F*_*m*_: maximal fluorescence when all PSII RCs are closed
*F*_*v*_/*F*_*m*_: maximum quantum yield of PSII photochemistry
NPQ: non-photochemical quenching of maximal fluorescence
PS: photosystem
qP: photochemical quenching of variable fluorescence
RCs: reaction centers
R_*Fd*_: fluorescence decline ratio under a given light condition
0 hS: non-shading control
3 hS: three hours of shading until 10:00
5 hS: five hours of shading until 12:00
7 hS: seven hours of shading until 14:00
VLICs: various light intensity conditions.
